# Neurosharing: large-scale data sets (spike, LFP) recorded from the hippocampal-entorhinal system in behaving rats

**DOI:** 10.12688/f1000research.3895.1

**Published:** 2014-04-30

**Authors:** Kenji Mizuseki, Kamran Diba, Eva Pastalkova, Jeff Teeters, Anton Sirota, György Buzsáki

**Affiliations:** 1 NYU Neuroscience Institute, Langone Medical Center, New York University, New York, NY, USA; 2 Center for Molecular and Behavioral Neuroscience, Rutgers, The State University of New Jersey, Newark, NJ, USA; 3 Allen Institute for Brain Science, Seattle, WA, USA; 4 Department of Psychology, University of Wisconsin at Milwaukee, Milwaukee, WI, USA; 5 Janelia Farm Research Campus, Howard Hughes Medical Institute, Ashburn, VA, USA; 6 Redwood Center for Theoretical Neuroscience, University of California, Berkeley, Berkeley, CA, USA; 7 Centre for Integrative Neuroscience, University of Tübingen, Tübingen, Germany; 8 Center for Neural Science, New York University, New York, NY, USA

## Abstract

Using silicon-based recording electrodes, we recorded neuronal activity of the dorsal hippocampus and dorsomedial entorhinal cortex from behaving rats. The entorhinal neurons were classified as principal neurons and interneurons based on monosynaptic interactions and wave-shapes. The hippocampal neurons were classified as principal neurons and interneurons based on monosynaptic interactions, wave-shapes and burstiness. The data set contains recordings from 7,736 neurons (6,100 classified as principal neurons, 1,132 as interneurons, and 504 cells that did not clearly fit into either category) obtained during 442 recording sessions from 11 rats (a total of 204.5 hours) while they were engaged in one of eight different behaviours/tasks. Both original and processed data (time stamp of spikes, spike waveforms, result of spike sorting and local field potential) are included, along with metadata of behavioural markers. Community-driven data sharing may offer cross-validation of findings, refinement of interpretations and facilitate discoveries.

## Introduction

The hippocampus and entorhinal cortex are essential structures for memory and spatial navigation ^1– 8^. Position-tuned cells (‘place cells’) are present in CA1, CA3 and dentate gyrus regions ^1, 9^. Grid cells, head direction cells, and border cells have been described in the dorsomedial entorhinal cortex, and are critical ingredients of navigation systems ^5, 7, 8, 10– 13^. The temporal coordination across the entorhinal cortex and hippocampus is secured by various oscillations, especially theta, gamma and sharp wave ripples ^14– 21^.

We recorded activity of neurons in these brain regions while animals performed various tasks, such as linear track, open maze, T-maze with wheel running delay, plus maze and zigzag maze, as well as recordings during sleep in the home cage. Extensive technical descriptions of the data sets described in this document are available in several published papers ^6, 21– 27^.

Several questions related to memory, navigation, spike time patterns, population coding, neuronal interactions, neuronal classification, replay, sleep homeostasis and oscillations have been studied based on this dataset ^6, 21– 41^. However, this dataset may provide valuable information if subjected to yet further analyses. Improved spike sorting, neuron classification and more sophisticated analyses may extend and refine the initial conclusions and offer insights that were previously missed. For these reasons we provide both unprocessed (wide band) and processed versions of our data. In our experience, all methods have limitations and must undergo continuous revision. We believe that community-driven data sharing, cross-validation of data, unified data formats and large collaborative efforts will facilitate discovery and benefit future progress in neuroscience.

## Material and methods

### Animal surgery

All protocols were approved by the Institutional Animal Care and Use Committee of Rutgers University (protocol No. 90-042), and all experiments were performed at Rutgers University. Before surgery, one to four rats were housed in a single home cage (made of plastic; size L = 45 cm, W = 23.5 cm, H = 20 cm). Wood shavings were used as bedding and dry pellets were provided as food. The animals were housed in a temperature controlled (68°F), but not a specific pathogen free, environment under 12:12-hours light:dark cycle where light cycle was from 7AM to 7PM. After surgery, the rats were housed individually, and highly absorbent paper (Techboard, Shepherd Speciality Papers) was used as bedding, and the animal’s health was assessed daily by the experimenters.

Details of surgery and recovery procedures have been previously described in detail ^42, 43^. Eleven Long Evans rats (male, 3–8 months old, 250–400 g) were deeply anesthetized with isoflurane (1–1.5%). In two rats (f01_m and g01_m), two silicon probes were implanted (one in each hemisphere) and targeted CA1 region. In three rats (gor01, pin01 and vvp01), two probes (32- and/or 64-site silicon probes) were implanted in the left dorsal hippocampus, targeted to CA1 and CA3 separately, and advanced over sessions and days through overlying neocortical and hippocampal tissue. The probe positions were: rat pin01: CA3: at a 35 degree angle to coronal plane, centered on 2.8 mm posterior and 2.6 mm lateral to bregma. CA1: 26.5 degree angle to vertical, at a 35 degree angle to coronal, centered on 4.6 mm posterior and 2.4 mm lateral to bregma; rat vvp01: CA3: at a 26.5 degree angle to coronal plane, centered on 2.8 mm posterior and 2.6 mm lateral to bregma. CA1: 26.5 degree angle to vertical, parallel to sagittal plane, centered on 4.4 mm posterior and 2.3 mm lateral to bregma; rat gor01: CA3: at a 26.5 degree angle to coronal plane, centered on 3.1 mm posterior, and 3.0 mm lateral to bregma. CA1: 26.5 degree angle to vertical, at a 45 degree angle to coronal plane, centered on 4.9 mm posterior and 1.5 mm lateral to bregma. In four rats (ec013, ec014, ec016 and i01_m), 32- or 64-site silicon probe(s) were implanted in the right dorsal hippocampus and recorded from CA1, CA3 or dentate gyrus, and another 4-shank silicon probe was implanted in the right dorsocaudal medial entorhinal cortex. In one rat (ec012), one 4-shank silicon probe was implanted in the right dorsocaudal medial entorhinal cortex. In rat ec012, ec013, ec014, and ec016, the probe targeting the entorhinal cortex was positioned such that the different shanks recorded from different layers ^21^ (4.5 mm lateral from the midline; 0.1 mm anterior to the edge of the transverse sinus at a 20–25 degree angle in the sagittal plane with the tip pointing toward the anterior direction). In rat i01_m, the EC probe had 4 shanks and was positioned such that all shanks recorded from the same layer. For the hippocampus probe in rats ec013, ec014 and ec016, the shanks were aligned parallel to the septo-temporal axis of the hippocampus (45 degrees parasagittal), positioned centrally at 3.5 mm posterior from bregma and 2.5 mm lateral from the midline.

For all silicon probes used, each shank had eight recording sites (160 µm ^2^ each site, 1–3-MΩ impedance), and intershank distance was 200 µm. Recordings sites were staggered to provide a two-dimensional arrangement (20 µm vertical separation) ^44, 45^. The individual silicon probes were attached to respective microdrives and moved independently and slowly to the target. Two stainless steel screws inserted above the cerebellum were used as indifferent (reference) and ground electrodes during recordings. At the end of the physiological recordings during the behavioural tasks, a small anodal DC current (2–5 µA, 10 s) was applied to recording sites 1 or 2 days before rats were deeply anesthetized and euthanized by perfusion with 10% formalin solution. The positions of the electrodes were confirmed histologically and reported previously in detail ^21, 24^.

### Behavioural testing

After the animals recovered from surgery (1 to 2 weeks), physiological signals were recorded during eight different types of behaviours mostly during light cycles (see Table 1).

**Table 1.** Behaviour descriptions.

**Behaviour**	**Behaviour subclass** **(Behaviour** **identifier)**	**Description**
elevated linear track	linear	Linear track, 250 cm × 7 cm.
elevated linear track	linearOne	Linear track (170 cm × 6.2 cm, with 22 cm × 22 cm end platforms) that was shortened to 79 or 125 cm in some trials ^23, 24^ (Usually shortened but sometimes also lengthened). The same linear track was used in linearOne and linearTwo but at different locations in the same recording room. The center of the track was at the same position for linearOne and linearTwo, but the track was at fixed 36.9 degree angles from each other, corresponding to the diagonals of the 480 × 640 pixel camera.
elevated linear track	linearTwo	Exactly the same as linearOne but the linear track was at different locations in the same recording room. See linearOne.
open field	bigSquare	180 cm × 180 cm.
open field	bigSquarePlus	180 cm × 180 cm square open field, divided by plus shaped walls put in the center of the field.
open field	midSquare	120 cm × 120 cm.
open field	Open	100 cm × 200 cm
rewarded wheel- running task	wheel	Operant wheel running task, See Mizuseki *et al.*, 2009 ^21^.
alternation task in T-maze	Mwheel	Alternation task in T-maze (100 cm × 120 cm) with wheel running delay. See Pastalkova *et al.*, 2008 ^6^
alternation task in T-maze	Tmaze	Alternation task in T-maze, the same as Mwheel but without delay period. There were 2.78 camera pixels/cm, which converts to 22.24 units/cm for the .whl files (8x compression of pixels).
elevated plus maze	plus	Plus maze. 100 cm × 100 cm.
zigzag maze	Zigzag	100 cm × 200 cm zigzag maze. See Royer *et al.*, 2010 ^46^.
wheel-running in home cage	wheel_home	Wheel running in home cage with free access to a wheel with no reinforcement.
sleep	sleep	Sleeping in home cage.

(1) On an elevated linear track (250 cm × 7 cm), the animals were required to run back and forth to obtain water reward at both ends ^21^. In three animals (gor01, pin01, and vvp01), a similar elevated track was used (170 cm × 6.2 cm, with 22 cm × 22 cm end platforms) that was shortened to 79 or 125 cm in some trials ^23, 24^.
(2) In the open field task, the rats chased randomly dispersed drops of water or pieces of Froot Loops (25 mg; Kellogg’s) on an elevated open platform ^21^ (180 cm × 180 cm, 120 cm × 120 cm or 100 cm × 200 cm).
(3) In the rewarded wheel-running task, a wheel (diameter = 29 cm) was attached to a rectangular-shape box (39 cm × 39 cm × 39 cm). The rat was required to run in the wheel continuously for 10 seconds, after which time a piece of Froot Loop was dropped in the box as reinforcement ^21^.
(4) In the alternation task in the T-maze (100 cm × 120 cm) with wheel running delay, the animal was required to run on a wheel attached to the waiting area for 10 sec, after which time the animal had access to the central arm of the T-maze, at the end of which the animal chose to turn right or left. The animal was rewarded with water if the choice was opposite to the previous one ^6^.
(5) In the elevated plus maze (100 cm × 100 cm), the rats were motivated to run to the ends of four corridors, where water was given every 30 s.
(6) In the zigzag maze (100 cm × 200 cm) with 11 corridors, the animals learned to run back and forth between two water wells; 100 µl of water was delivered at each well ^21, 22, 25, 46^.
(7) In the wheel-running in home cage, a wheel (diameter = 29 cm) was attached to a rectangular-shape box (39 cm × 39 cm × 39 cm) which was used as a home cage during the experiment. Rats had free access to the wheel, and ran on the wheel with no reinforcement.
(8) In the sleeping session, the rat slept in the home cage.

For recording of behaviour (1) to (6), animals were water-scheduled for 23 hours prior to the experiment. Otherwise, both dry food and water were provided ad libitum. For tracking the position of the animals, two small light-emitting diodes, mounted above the headstage, were recorded by a digital video camera (SONY) at 30 Hz resolution.

### Data collection and cell-type classification

Detailed information about the recording system and spike sorting has been previously described ^21, 24, 42^. Briefly, signals were amplified (1,000×), bandpass-filtered (1 Hz–5 kHz) and acquired continuously at 20 kHz (DataMax system; RC Electronics) or 32,552 Hz (NeuraLynx, MT) at 16-bit resolution. After recording, the signals were down-sampled to 1,250 Hz (DataMax system) or 1,252 Hz (NeuraLynx system) for the local field potential (LFP) analysis. In electrophysiological recordings, positive polarity is from zero toward positive values. To maximize the detection of very slowly discharging (‘silent’) neurons ^47^, clustering was performed on concatenated files of several behavioural and sleep sessions recorded at the same electrode position on the same recording day ^22, 25– 27^. We made extensive use of publicly available analytical and display programs, which were developed in our laboratory (KlustaKwik ^48^ available at http://sourceforge.net/projects/klustakwik/, Neuroscope ^49^ available at http://neuroscope.sourceforge.net/, Klusters ^49^ available at http://klusters.sourceforge.net/, NDmanager ^49^ available at http://ndmanager.sourceforge.net/). The latest available version at the time was used in each case. Spike sorting was performed automatically, using KlustaKwik ^48^, followed by manual adjustment of the clusters, with the help of autocorrelogram, cross-correlogram and spike wave-shape similarity matrix (Klusters software package ^49^). Because none of the existing spike sorting algorithms is completely automated, manual adjustment is necessary ^48^. This inevitably leads to some operator-dependent variability ^48^; therefore, provided clusters are not always identical to those used in our previous publications. Hippocampal principal cells and interneurons were separated based on their burstiness, waveforms and short-term monosynaptic interactions ^6, 17, 21, 24, 42^. Classification of principal neurons and interneurons of entorhinal cortical neurons was based on waveforms and short-term monosynaptic interactions, and described previously in detail ^21^. A total of 3,113 (CA1), 882 (CA3), 66 (DG), 491 (EC2), 568 (EC3) and 551 (EC5) principal neurons and 420 (CA1), 198 (CA3), 52 (DG), 85 (EC2), 215 (EC3) and 91 (EC5) interneurons were identified and included in this data set (see Table 2– Table 4).

**Table 2.** Number of cells recorded. Top row: animal identifier. Left column: brain region. Brain region EC4 indicates either entorhinal cortex layer 3 or 5 (could not be determined which); region EC? indicates in entorhinal cortex, but without layer assignment.

Brain region	ec012	ec013	ec014	ec016	f01_m	g01_m	gor01	i01_m	j01_m	pin01	vvp01	total
EC2		311	180	112								603
EC3	201	362	177	116								856
EC4		276		57								333
EC5	110	416	68	154								748
EC?								82				82
Total EC	311	1365	425	439				82				2622
												
CA1		1185	1136	661	99	145	50	309		23	116	3724
CA3		223		646			153			45	56	1123
DG		41		94								135
Unknown		39						3	90			132
Total	311	2853	1561	1840	99	145	203	394	90	68	172	7736

**Table 3.** Number of principal cells. Top row: animal identifier. Left column: brain region.

Brain region	ec012	ec013	ec014	ec016	f01_m	g01_m	gor01	i01_m	j01_m	pin01	vvp01	total
EC2		248	146	97								491
EC3	140	239	101	88								568
EC4		214		46								260
EC5	89	300	34	128								551
EC?								51				51
Total EC	229	1001	281	359				51				1921
												
CA1		887	995	577	79	131	42	289		19	94	3113
CA3		217		443			138			41	43	882
DG		18		48								66
Unknown		37						1	80			118
Total	229	2160	1276	1427	79	131	180	341	80	60	137	6100

**Table 4.** Number of interneurons. Top row: animal identifier. Left column: brain region.

Brain region	ec012	ec013	ec014	ec016	f01_m	g01_m	gor01	i01_m	j01_m	pin01	vvp01	total
EC2		45	27	13								85
EC3	37	89	66	23								215
EC4		31		8								39
EC5	16	36	20	19								91
EC?								24				24
Total EC	53	201	113	63				24				454
												
CA1		205	90	46	19	13	8	14		3	22	420
CA3		4		174			14			2	4	198
DG		16		36								52
Unknown		1						1	6			8
Total	53	427	203	319	19	13	22	39	6	5	26	1132

**Table 5.** Number of recording sessions. Top row: animal identifier. Left column: behaviour subclass.

Behaviour subclass	ec012	ec013	ec014	ec016	f01_m	g01_m	gor01	i01_m	j01_m	pin01	vvp01	total
bigSquare	24	45	4	13				1	4			91
bigSquarePlus		2										2
linear	18	90	2	9								119
linearOne							3				5	8
linearTwo							3				5	8
midSquare		4	8	2								14
Mwheel	28	16	8	14	8	7		8				89
Open											3	3
plus		11										11
sleep			19	10							1	30
Tmaze							2			3	1	6
wheel		40	8	9			1					58
wheel_home				2								2
ZigZag			1									1
Total	70	208	50	59	8	7	9	9	4	3	15	442

The tip of the probe either moved spontaneously relative to the brain or was moved by the experimenter between recording days to record from potentially different sets of neurons. However, we cannot exclude the possibility that some neurons recorded on different days were identical, because spikes recorded on each day were clustered separately, though in some instances neurons were recorded over multiple days. When we moved the electrodes, we waited for at least an hour before recording in order to stabilize the position of electrodes.

### Data description

The data are available ^50^ at CRCNS.org ( http://dx.doi.org/10.6080/K09G5JRZ). Details of the data collection, processing and storage of data into files are included with the data set, including scripts useful for processing the data ^50^. Here, we briefly summarize the data description.

The number of cells recorded from each animal and brain region is shown in Table 2.

Most of the recorded cells were classified as principal neurons or interneurons. The number of cells classified as principal and interneuron are shown in Table 3 and Table 4.

The 8 types of behaviours (see Behavioural Testing section) were further subdivided into 14 behaviour subclasses based on minor differences (e.g. size of maze) and used as behaviour identifiers in the dataset ( Table 1).

The data were obtained during 442 recording sessions. During each session the animal performed one of the 14 behaviour subclasses. The number of recording sessions and behaviour subclasses used with each animal is shown in Table 5. The description of each behaviour subclass is given in Table 1.

### Data file organization

The data files for each recording session are stored in separate compressed tar archive files (i.e. with extension “tar.gz”). These files are organized into top-level directories, each of which contains data for sessions recorded on the same day using the same animal and electrode placement combination. Data from all sessions recorded from the same animal on the same day were merged for spike sorting. All merged sessions are stored in the same top-level directory in the data set at CRCNS.org. Therefore, the cell identification numbers assigned by the spike sorting are common to all sessions within a top-level directory, and are not specific to individual sessions. Details of the file organization are provided in the document “CRCNS.org hc3 data description” which is included with the data set.

### Metadata organization

The metadata describing the data is stored in four tables that are included with the data set. Table *cell* has information about each spike sorted cell. Table *session* has information about each experimental session. Table *epos* contains information about the position of the electrodes. And table *file* has information about the “.tar.gz” and other files that are in the data set.

These tables are provided in CSV (comma-separated values) format, Excel format, and as tables in an SQLite database. SQLite ( http://www.sqlite.org/) is a free, open source, SQL data base engine available for all common operating systems. These tables are related to each other through a field (named “topdir”), which has the name of top-level directories described above and is common to all four tables. The fields in each of these tables are listed in Listing 1. As described in file “CRCNS.org hc3 data description” the SQLite command interface can be used with these tables to generate summary statistics from the metadata and to locate data files that satisfy particular search criteria (for example, find data for cells of a specific type from a particular brain region and experimental behaviour).

**Listing 1: Create table statements for tables: cell, session, file and epos.** Fields for each of these tables are documented in the comments.


create table cell
 id integer,       -- Id used to match original row number in MatLab PyrIntMap.Map matrix
 topdir string,    -- top level directory containing data
 animal string,    -- name of animal
 ele integer,      -- electrode number
 clu integer,      -- ID # in cluster files
 region string,    -- brain region
 nexciting integer,   -- number of cells this cell monosynaptically excited 
 ninhibiting integer, -- number of cells this cell monosynaptically inhibited 
 exciting integer,    -- physiologically identified exciting cells based on CCG analysis
 inhibiting integer,  -- physiologically identified inhibiting cells based on CCG analysis
         -- (Detailed method in Mizuseki Sirota Pastalkova and Buzsaki., 2009 Neuron paper.)
 excited integer,     -- based on cross-correlogram analysis, the cell is monosynaptically 
excited by other cells
 inhibited integer,   -- based on cross-correlogram analysis, the cell is monosynaptically 
inhibited by other cells
 fireRate real, -- meanISI=mean(bootstrp(100,'mean',ISI)); fireRate = SampleRate/MeanISI; ISI is 
interspike intervals.
 totalFireRate real,  -- num of spikes divided by total recording length
 cellType string      -- 'p'=pyramidal, 'i'=interneuron, 'n'=not assigned as pyramidal or 
interneuron
);

create table session (
  id integer,     -- matches row in original MatLab Beh matrix
  topdir string,  -- directory in data set containing data (tar.gz) files
  session string, -- individual session name (corresponds to name of tar.gz file having data)
  behavior string, -- one of: Mwheel, Open, Tmaze, Zigzag, bigSquare, bigSquarePlus,
                   -- linear, linearOne, linearTwo, midSquare, plus, sleep, wheel, wheel_home
  familiarity integer, -- number of times animal has done task, 1=animal did task for first time, 
                       -- 2=second time, 3=third time, 10=10 or more
  duration real   -- recording length in seconds
);

create table file (
  -- information about files in hc3 dataset
  topdir string,  -- directory in data set containing data (tar.gz) files
  session string, -- individual session name (corresponds to name of tar.gz file having data)
  size integer,   -- number of bytes in tar.gz file
  video_type string, -- 'mpg', 'm1v' or '-' (for no video file)
  video_size integer -- size of video file, or 0 if no video file
);

create table epos (
  -- has electrode positions for each top level directory
  -- Note, some regions do not match that in cell table.
  -- Those that differ have following meanings:
  --   DGCA3: not sure if the electrode is DG or CA3.
  --   Ctx: somewhere in the cortex (above the hippocampus)
  --   CA: somewhere in the hippocampus (do not know if it is CA1, CA3 or DG)
  topdir string,  -- directory in data set containing data (tar.gz) file
  animal string,  -- animal name
  e1 string,      -- region for electrode 1 
  e2 string,      -- region for electrode 2
  e3 string,      -- region for electrode 3
  e4 string,      -- region for electrode 4
  -- ... (e5 through e14 fields not shown)
  e15 string,     -- region for electrode 15
  e16 string      -- region for electrode 16
);



## Data availability

CRCNS: Multiple single unit recordings from different rat hippocampal and entorhinal regions while the animals were performing multiple behavioral tasks, http://dx.doi.org/10.6080/K09G5JRZ

Terms of data usage: Data on this site is made available only for scientific purposes. Redistribution of the data is not permitted. Any publications derived from the data must cite the data contributors and CRCNS.org as being the source of the data and the original paper(s) that generated the data. Unnecessary downloading of large data files is not permitted. (To minimize demands on the server, only data expected to be useful for your scientific purposes should be downloaded).

Privacy notice: Occasionally the researchers who contribute data wish to know who has downloaded their data. Upon request we will provide this information to the data contributors. So, if you download data, there is a possibility that your name and email address will be provided to the data contributor. We request that the data contributors only use the information for legitimate scientific purposes (such as determining the frequency of downloads, or contacting users to providing updated information about the data or to explore possible collaborations).
