# Supplementary material for: Neurosharing: large-scale data sets (spike, LFP) recorded from the hippocampal-entorhinal system in behaving rats
Source: F1000Res. 2014 Apr 30;3:98. [Version 1] doi: 10.12688/f1000research.3895.1 (PMC4097350; doi:10.12688/f1000research.3895.1)
Supplement: Peer review history

# Peer review: Referee response for version 1

**DOI:** 10.5256/f1000research.4171.r4603

**Published:** 2014-05-07

**Authors:** James Knierim

**Affiliations:** 1 Zanvyl Krieger Mind/Brain Institute and Department of Neuroscience, Johns Hopkins University, Baltimore, MD, USA

This data set will be a valuable resource for investigators who wish to test hypotheses about hippocampal function and interaction with entorhinal cortex at the level of single unit and LFP physiology. I have not investigated the data base carefully to ensure its utility, as I assume the authors have done so. My comments are limited to a few questions about their *F1000Research* article describing the data base.

1. *'Animal surgery' - *second paragraph:
  Do the authors mean "in the coronal plane?" It is not clear whether the tetrodes were angled medially or laterally in that plane, or whether the authors mean that the tetrodes were angled anteriorly or posteriorly to the coronal plane. Please clarify here and in other locations in the text how the tetrodes were angled.
2. *'Animal surgery' - *final paragraph:
  Does the data base contain histological figures? If not, are they easily identifiable and accessible from published reports? It would be very useful to ensure that the precise location of tetrodes could be made available to investigators.
3. Table 1, ' *elevated linear track*':
  Shouldn't the fixed angles be 74 degrees (i.e. +/- 37 degrees)?
4. ' *Behavioural testing*'  item (5):
  Were the rats motivated to run the corridors in any particular order?  Was there any working memory component to the trials?

I have read this submission. I believe that I have an appropriate level of expertise to confirm that it is of an acceptable scientific standard.

# Peer review: Referee response for version 1

**DOI:** 10.5256/f1000research.4171.r4604

**Published:** 2014-05-06

**Authors:** Andrew P. Maurer

**Affiliations:** 1 Division of Neural Systems Memory and Aging, University of Arizona, Tucson, AZ, USA

Mizuseki and colleagues provide a description of 442 datasets (more than 200 hours) of hippocampal *in vivo* recordings. These datasets provide cell classification as well as the raw data, in case users wish to return to the high-sample traces and re-cluster the data on their own. The results of different analyses from the database have been published, although all analysis possibilities have not been exhausted. While this is an atypical review to write (as any suggestions on improvement to the database seem to be akin to looking a gift horse in the mouth), I am hoping the authors can discuss the ramifications of providing such an extensive database. While any database has limitations (e.g. sometimes determining the EEG units is a bit of an exercise with these data in the present format), it is perhaps more advantageous and constructive to discuss what the community can do to make the most use of it. As the authors state *“Community-driven data sharing may offer cross-validation of findings, refinement of interpretations and facilitate discoveries”* and the challenges are now directed to those who are interested in participating in future analyses. In hopes that this will set-forth a new era of data-sharing, I hope that the authors can discuss their open database in a manner that parallels Giorgio Ascoli’s discussion on sharing neural reconstruction files (Ascoli, 2006). It should be noted that I do not expect the authors to have comprehensive answers to each of my comments below, but it might be beneficial if they would provide some initial thoughts to seed further discussion. Some points that the authors may wish to discuss include:

1. One barrier to sharing data is “the fear of being scooped” (Ascoli, 2006). For example, scientific progress will be dramatically increased through parallel (and hopefully, collaborative) data analysis. Are the authors concerned about being “scooped”? What about a group of researchers unknowingly using the database to conduct an analysis that is also a student’s PhD project? This hypothetical student may still be acquiring the expertise to keep pace with more seasoned researchers.
2. “An often unspoken resistance to the sharing… data is born out of concern for criticisms and mistakes” (Ascoli, 2006). This is an unprecedented event that Mizuseki and colleagues have set forth, by providing a comprehensive catalog of data in an unabashed manner. The first point I want to touch upon is the level of raw exposure in releasing data. I have never met a neuroscientist who believes that anyone else could’ve conducted their analyses better than themselves (save a modest few), but to find those who believe that the authors “didn’t look for the right thing” could make up a small battalion. Mizuseki and colleagues invite critics to their doorstep. Perhaps this is more similar to posing nude as a model for artist. By placing data online, it comes with judgment and the potential to be proved wrong. What do the authors believe the convention is if others follow in their footsteps? For example, if group A shares their database after publishing their results, group B downloads and analyzes their data in new light and finds the opposite results, what is the appropriate manner in handling the situation? The self-correction aspect of science is also accelerated when data is openly-shared. It remains to be seen how situations like this should/will be handled. 
3. There is also a chance for the data to be used in order to stifle or impede publication when the result is dubious. That is, should it be considered “fair-play” for a reviewer to use the same database and conduct a similar analysis with results that contradict a submitted manuscript’s results? A tactic such as this only seems appropriate in “open-review formats”.
4. “A final barrier to sharing digital reconstructions relates to the reluctance to lose or give a competitive edge” (Ascoli, 2006). The release of this immense database will surely be the stronghold of many new assistant professors who are still in the initial stages of setting up a physiology laboratory. Moreover, I can see these data being used in laboratories that are heavily analysis driven and limited in their own capacity for high-density *in vivo* recordings. This increase in the number of people analyzing data invites competitors for the authors as well as their neutral peers. Sheer logic dictates that the authors are not afraid of competition (otherwise, why share the data? Please note that I am a cynic and have been taught by many reviewers that “scientific altruism” is as abundant as snarks and unicorns). Why should researchers not be afraid of others using this database to compete?  Is it believed that these data will be used for collaboration? What can be done to emphasize collaboration across laboratories when using the database? How is authorship handled when multiple groups use these data? If I spend my time analyzing these data, only to have it published under a “group project name” (similar to Sir William Timothy Gowers’ Polymath project), do I put it on my CV?
5. The paper explicitly states that large downloads are prohibited. Does this mean that I should not download all the data? Is it OK to use these data for a class project? If so, is it more appropriate for the professor to disseminate it to the students or should the students make their own CRCNS account? Finally, more of an afterthought but along similar lines: should there be a collaborative processing code library that should be developed and maintained in parallel with the use of these data (similar to GitHub or SourceForge)? 

 

I absolutely do not expect the authors to have complete answers to these questions nor should they carry the sole responsibility of determining the general conventions of what constitutes use versus misuse, but I do think that it is worth hearing their general thoughts. As this is the largest and most comprehensive database of *in vivo* hippocampal and entorhinal physiology to become available to the general scientific community, there will be an immense ramification. Scientific replication/external validation is an immediate and positive application of this database. As I have cited Timothy Gowers above, I think it would be best to leave off with his opinion of open data-sharing and collaboration: “It feels as though this process is to normal research as driving is to pushing a car.” ( http://gowers.wordpress.com/2009/02/01/questions-of-procedure/).

As a field we have the opportunity to compete or collaborate. I hope that these data facilitate cross-laboratory collaboration where two groups are reticent to share their own data. For those that are interested in embracing the collaborative spirit, the CRCNS website also has a “marketplace” section where ideas and potential collaborations can be discussed. Finally, I applaud the authors for this unprecedented act of scientific altruism. I hope this will be a platform that accelerates our understanding of the entorhinal-hippocampal circuitry through collaboration.

I have read this submission. I believe that I have an appropriate level of expertise to confirm that it is of an acceptable scientific standard.

# Peer review: Referee response for version 1

**DOI:** 10.5256/f1000research.4171.r4607

**Published:** 2014-05-06

**Authors:** Shuzo Sakata

**Affiliations:** 1 Strathclyde Institute of Pharmacy and Biomedical Sciences, University of Strathclyde, Glasgow, UK

This is an important report for the neuroscience community regarding huge data sets recorded from the dorsal hippocampus and dorsomedial entorhinal cortex of behaving rats. Because the authors have already published a number of papers with the data sets, I have no doubt that this paper and their shared data will contribute to the further advancement of this field.  

This manuscript is clearly written in details. This article can be improved by clarifying the following minor points in the section Data collection and cell-type classification:

**1. Cluster quality**

It is not clear whether the quality of single units was assessed objectively, for example, by measuring isolation distance.

**2. Cell-type classification**

Because now researchers including the authors can classify cell-types optogenetically, it would be better to comment on potential pitfalls of the classification procedure used here. For example, it is difficult to exclude the possibility that some of “principal” neurons can be interneurons.

I have read this submission. I believe that I have an appropriate level of expertise to confirm that it is of an acceptable scientific standard.

# Peer review: Referee response for version 1

**DOI:** 10.5256/f1000research.4171.r4602

**Published:** 2014-05-06

**Authors:** Yoshikazu Isomura

**Affiliations:** 1 Brain Science Institute, Tamagawa University, Tokyo, Japan

In this work, Mizuseki *et al.* provide valuable information on their large-scale data sets of multi-neuronal and local field potential (LFP) recordings from the hippocampus (dentate gyrus, CA3, and CA1) as well as the enthorhinal cortex across the layers (layers 2, 3, 4, 5 etc.) of behaving rats. These data sets have originally been used for a number of their excellent studies for past ten years. Here, they fully summarized the experimental conditions and results in individual recording sessions very clearly for any users to plan to analyze the data.  It will be a really useful treasure map for all hippocampus researchers.

I have one minor comment. While they showed the number of isolated neurons in each brain area of each animal (Table 2 to Table 4), it is not clear yet how many neurons, at most, were recorded simultaneously.  Some people will probably want to analyze spike activity in a population of many neurons. The data would be more available for the higher-order spike analysis if the authors added additional information on the best (or first choice) data set with a sufficient number of well-isolated neurons in the same brain area.

I have read this submission. I believe that I have an appropriate level of expertise to confirm that it is of an acceptable scientific standard.
